# Demographic, Personality and Psychopathology Characteristics of the Runaway Girls in Social Emergency and Rehabilitation Centre of Shiraz, Iran

**Published:** 2012

**Authors:** Shahin Toubaei, GholamReza Nateghi, Gholam Reza Dehbozorgi, Hasan Sadr Esfahani

**Affiliations:** 1 Department of Psychiatry, Shiraz University of Medical Sciences,; 2 Psychiatric and Behavioral Sciences Research Center, and Department of Psychiatry, Mazandaran University of Medical Sciences, Sari, Iran.; 3 Psychiatrist

**Keywords:** personality disorder, physical abuse, psychiatric disorder, psychopathology, Runaway girls

## Abstract

**Objective**: The problem of runaway girls is one of the social problems which has become more prevalent and is considered a serious challenge for families, welfare centers and governmental organizations in Iran. This study aimed at determining the demographic, personality and psychopathology characteristic of a sample of runaway girls in Shiraz, Iran.

**Methods**: Fifty girls who had escaped from their home and were referred to the Women’s Social Emergency and Rehabilitant Centre of Shiraz were compared with fifty girls who lived with their parents (control group). They were assessed by semi-structured interview based on the DSMIV-TR criteria, demographic questionnaire, the General Health Questionnaire-28 (GHQ-28) and the Eysenck Personality Questionnaire-R-106 (EPQ-R-106).

**Results: **Mean (±SD) age of the runaway girls was 19.9 (±3.81) years. Twenty (40%) were the first child of the family. Forty-three subjects (86%) were resident of cities. Physical abuse and neglect were more prevalent in the runaway girls (P < 0.05). There were no significant differences between two groups in history of major psychiatric disorders. Regarding GHQ-28, only in social function subscale, the runaway girls showed more disturbances in their social function compared to control group (P < 0.05). There were significant differences in extroversion, lying tendency, addiction tendency and crime seeking (P < 0.05) between the two groups and escaped girls showed more disturbances in comparison with the control group.

**Conclusion**: The social burden of runaway girls in Shiraz is of significance and this subject warrants more attention from non-governmental and governmental organizations in order to provide more psychological and social support for these girls.

## Introduction

According to previous studies, running away in adolescents associated with substantial emotional impairment (1). Street children appeared as a major concern since international year of child 1979, and then became focus of attention for welfare agencies and government (2)

The United Nations defines a street youth as “any boy or girl for whom the street (in the widest sense of the word, including unoccupied dwellings, wasteland, etc.) has become his or her habitual abode and/or source of livelihood; and who is inadequately protected, supervised, or directed by responsible adults” (3). Majority of street children in researches are male. In Latin America, where half of street children of world were found (4), studies reported more running away in boys than girls (5).

In recent years, however, the number of girls on the streets has increased and attempts to work with female street youth are hampered by lack of knowledge about how the experiences of girls on the street differ from those of their male peers (6-8).

Although cultures differ in specific behaviors that are considered acceptable for males and females, there are certain similarities that are relevant to the situation of homeless youngsters. In most of the world’s cultures, women and girls are traditionally kept close to home because of child-care and family responsibilities and for their own protection, whereas men and boys are encouraged to go out of the home for recreation and to earn a living. As a result of this separation of the masculine and feminine spheres, in many cultures street girls appear to be more “out of place” than street boys (7, 9).

Because the presence of girls on the streets violates cultural norms for female behavior, it has been proposed that homeless girls are more likely to be from dysfunctional families and exhibit psychological distress than their male peers (10, 11). In impoverished families, presence of boys in street might reflect a deliberate strategy of life for making independent men; in contrast, street girls usually reflect family dysfunction. Then, street girls had poorer outcome relative to boys because of disruption of social norms (10, 11).

Some social factors like urban-to-rural migration, high birth rates, economic stagnation, and lack of government welfare programs are attributed to children and adolescents living/working on the streets in Latin America. (4, 12-14).

In fact, there are a large number of impoverished families in Latin America; but it is unlikely that poverty alone can be reason of presence of adolescents on the street (15).

 (15). A review study conducted in Latin America indicated that impoverished youngsters of homeless families are more troubled than those impoverished adolescents working on streets but living at home; and more homeless than working youth experienced physical abuse, parental absence or death, and rural-to-urban migration prior to leaving home. Raffaelli concluded in this study that family disruption is an important factor in the street youth phenomenon, and having a place for living might be more important than poverty (16). 

The results of a studyof 120 girls selected from three groups in Tehran showed significant differences between runaway and non-runaway groupsin negative emotional-focused coping styles. Runaway girls scoredsignificantly higher in negative emotional-focused coping styles.However, there was not any significant difference between positiveemotional-focused and problem-focused coping styles of runaway andnon-runaway girls (17). 

This study was performed to assess the demographic, personality and psychopathology characteristics of girls who escaped from their home in Shiraz, the capital of Fars province in Iran

## Methods and Materials

This study was carried out in 2007 in Shiraz, Iran. Fifty runaway girls referred by police and their families because of runaway and maladaptive behaviors to the Women Social Emergency and Rehabilitation Centre (WSERC) of Shiraz were compared with fifty girls who lived with their parents (control group).

**Table 1:** Comparison of demographic characteristics between runaway girl and control group in Shiraz, Iran

	**Groups**	**Mean**	**SD**	**t**	**P**
Age(years)	Escaped Not escaped	19.9 20.04	3.8 6.3	0.135	0.89
Education, (Years)	Escaped Not escaped	7.94 8.84	3.04 2.5	1.25	0.21
Family income (Rials per month)	Escaped Not escaped	1320000 1410000	134362 127827	0.36	0.71
Father’s education (Years(	Escaped Not escaped	4.95 4.7	4.33 3.83	0.302	0.76
Mother’s education (Years(	Escaped Not escaped	4.36 4.4	3.43 4.01	0.04	0.96
Number of family Members	Escaped Not escaped	6.8 6.54	2.22 2.2	0.58	0.58

The WSERC is a dormitory providing accommodation and psychological supports to women and girls with a positive history of disruptive behaviors and running away experience. The control group was selected from students of Shiraz schools. Two groups were matched for age, education, and economic status, number of family and literacy level of their parents. Their demographic characteristics are summarized in table 1. Both groups filled out the informed consent form.

Each girl was asked to complete a demographic questionnaire, an Iranian form of the General Health Questionnaire-28 (GHQ-28) and the Eysenck Personality Questionnaire-R-106 (EPQ-R-106) anonymously. Also, they were evaluated by a semi-structured interview based on fourth edition of the American Psychiatric Association Diagnostic and Statistical Manual of Mental Disorders (DSM-IV TR). GHQ-28 is a 28 item self-report questionnaire designed for screening mental disorders in different contexts by Goldberg in 1972. The questionnaire thus attempts to measure four dimensions of psychopathology including somatization, anxiety, social dysfunction and depression. GHQ is the most recognized instrument for screening mental disorder in wide researches in the world (18). Test-retest reliability for Iranian population was reported to be 0.91 (18). Using traditional GHQ score by Likert scale, a global score equal or greater than 23 indicates poor general health in the past month (19).

EPQ-R-106 is a self-report questionnaire with 106 items devised to measure three major personality dimensions including psychoticism, extroversion and neuroticism. It contained a lie scale to alert the investigator to dissimulation and tendency to addiction. Psychometric characteristics of EPQ showed a high and acceptable reliability for it: the reliability was 0.86 for tendency to addiction, 0.88 for crime seeking, 0.92 for extroversion, 0.88 for lie scale, 0.89 for neuroticism and 0.72 for psychoticism (20). The test-retest reliabilities were quite satisfactory ranging from 0.80 to 0.86. Barrett and Eysenck reported the means of three personality traits of extraversion, neuroticism, and psychoticism for 37 nations including Iran (21, 22).

*Clinical diagnosis*

After completing the above mentioned questionnaires, for assessment of their psychiatric disorders the girls were interviewed by two psychiatrists separately. If there was an agreement between them, the diagnosis of psychiatric disorder of interest was made. 

*Statistical analysis*

The data were analyzed by the SPSS software for Windows (ver. 10.0) using univariate analysis of variance and multivariate analysis.

## Results

Mean (±standard deviation, SD) age of the runaway girls was 19.9 (±3.81) years. Among 50 girls who escaped from their home, 20 (40%) were the first child, 7 (14%) the second child, 12 (24%) the third child, 5 (10%) the fourth child, 3 (6%) the fifth child, 2 (4%) the sixth child and 1 (2%) the seventh child. Forty-three subjects (86%) were resident in cities and 7 (14%) were villagers. 

Twenty-four fathers and seven mothers of escaped were reported to have drug addiction while in control group this condition was reported in five fathers and one mother. Also regarding family stability, 41 subjects had problems such as divorce of the parents, father being in prison, being neglected in their childhood, etc. while in control group nine subjects had experienced death of father and one person reported death of her mother (χ^2 ^= 0.83); (Table 2). 

**Table 2:** Comparison of family stability parameters between runaway and control girls in Shiraz, Iran

	Escaped	Control	Total	p
Divorce and death of parents	41	10	51	0.0001
Drug addiction in parents	31	6	37	0.0001

Physical abuse/neglect and psychiatric disorders were more prevalent in escaped girls (P < 0.05); (Table 3).

**Table 3:** Comparison of history of psychiatric disorders and physical abuse between runaway and control girls in Shiraz, Iran

	Escaped	Control	Total	P
Psychiatric disorder	23	2	25	< 0.0001
Physical abuse and neglect	48	21	69	< 0.0001

The profile of psychiatric disorders is shown in table 4. 

**Table 4:** Distribution of current psychiatric disorders in runaway girls and control group according to psychiatric interview

Frequency	Escaped		Control		P
Diagnosis	N	%	N	%	
Major Depressive Disorder	8	16	2	4	0.0956
Bipolar I Disorder	3	6	-	-	0.241
Schizophrenia	2	4	-	-	0.4751
Anxiety Disorders	2	4	-	-	0.4751
Antisocial Personality Disorder	2	4	-	-	0.4751
Histrionic Personality Disorder	2	4	-	-	0.4751
No psychiatric disorder	31	64	42	96	0.0002
Total	50	100	50	100	

Table 5 summarizes the result of GHQ in the two groups. Only in social function subscale, there were significant differences between the two groups and the escaped girls showed more disturbances in their social function compared to control group (T = 2.9, P < 0.05).

**Table 5:** Mean (SD) of GHQ-28 of escaped vs. non-runaway girls

Frequency	Escaped		Non Escaped			
Scales	Mean	%	Mean	%	T	**P**
**Somatization**	6.6	3.54	6.28	4.71	0.38	**0.7**
Anxiety	7.04	3.94	6.36	5.08	0.74	0.45
Social Function	8.82	2.91	7.04	3.21	2.9	0.005
Depression	5.7	4.69	4.6	4.84	0.83	0.4
Total	28.16	11.79	24.58	14.88	1.33	0.18

Table 6 summarizes comparison of the results of EPQ-106 in the two groups. There were significant differences in extroversion, lying tendency, addiction tendency and crime seeking (P < 0.05) between the two groups and escaped girls showed more disturbances in comparison with the control group. In psychoticism and neuroticism, there were not any significant differences between the two groups.

**Table 6:** Mean (SD) of EPQ-106 in escaped vs. non-runaway girls

Frequency	Escaped			Non Escaped		
Scales	Mean	SD	Mean	SD	T	P
Extroversion	12.22	3.9	14.84	3.19	3.67	<0.01
Psychoticism	9.76	3.76	8.56	3.97	1.54	0.12
Neuroticism	17.62	4.21	16.78	4.14	1.004	0.2
Lie Scale	12.94	3.5	11.04	3.75	2.61	0.01
Tendency to addiction	15.98	4.25	14.18	3.92	2.2	0.02
Crime seeking	20.04	4.04	18.1	4.13	2.37	0.02

## Discussion

Mean age of street children in India and Ethiopia has been reported to be 10-14 years (23, 24). The average age of some children in Ethiopia were reported by the United Nations Children's Fund UNICEF to be 11 years and in the studies done in developed countries, mean age of half of them was reported to be 16 years. It seems that higher mean age observed here compared to other reports is due to the study design which recruited the subjects from Women Social Emergency Center of Shiraz. So, due to this limitation the results are not comparable with other researches properly. As mentioned before, this center provides accommodation and psychological supports to girls who have at least 15 years old with disruptive behaviors and running away experience. A study conducted by Janus et al. noted that female runaways have a tendency to run away at an earlier age than male runaways (25).

Birth order of the escaped girls showed that 40% of them were the first child of the family. It may be explained by the fact that in our culture the first child suffers more than other children and is considered as the substitute of the parents at home. 

The runaway adolescent is viewed as serving three functions within her family. First, she often parents her parents and siblings. Second, she protects her parents' marriage and regulates marital distance. Third, she preserves her family unit at the preadolescent developmental stage. Interventions are described that remove the adolescent from those roles by empowering the parents to take charge of the adolescent, by changing the communication process such that the couple deal with their marital issues without the help of the teenager, and by facilitating the family's movement toward a new stage of separation and individuation (26).

Reported family incomes of the runaway girls (1320000±134362 Rials which are less than 150 US Dollars) indicate that they are from poor families, and is in agreement with the reports of Aptekar et al. in Nairobi (11). Furthermore, 49% of their fathers and 14.1% of their mothers had drug addiction whereas 10% of fathers and 2% of mothers of the control group had this condition. It can be concluded that drug addiction of parents which has detrimental effects on economic status of the family is one of the important factors responsible in escape of the girls from their home. 

History of physical abuse and psychiatric disorder were significantly high in the runaway girls. A high proportion (95.9%) of the escaped girls complained of physical abuse in their home, whereas 42% of the control group reported physical abuse. 

Lalor reported that 69% of girls escaped from home due to their familial physical abuse (27). These results were closer to the results of this study and probably one of the effective causes of escaping of the girls from home is the feeling of insecurity in their family.

In the runaway girls group, 46% reported psychiatric illnesses based on the DSM-IV TR criteria in their past history. Familial stability showed more disturbances than the control group; 22% of their parents had divorced, 28% of their fathers and 2% of their mothers were dead, and 2% of their fathers were prisoner and these results were compatible with other researches (28). 

It is reported that street children came from atypical families and 78% of them were from single parents or no parent family. Also, in UNICEF study in Ethiopia, 23% of street children lived with both parents and in Brown's study in Jamaica, 7% lived with their both parents. According to Andres-Lemay (1) physical and sexual abuse significantly increased the risk of running away (1). Adolescents with PTSD also were more likelyto have runaway (29).

If we look at the conditions that lead to a *career in the streets* and the pre histories of 'street children', it becomes obvious, that the *danger of a street career* frequently shows up very early for girls and boys who are neglected or abused by their parents or step-parents, because these youngsters often grew up in patchwork families with a lot of problems like drug or alcohol addiction, illness, poverty, etc (30).

The results of GHQ-28 test showed that the escaped girls reflect more disturbances in their social function so social skill training seems necessary for them. Also the EPQ-106 results showed that with a view to extroversion, lie-detection, addiction and crime seeking, there were significant differences between them and the control group, so these factors can bring about more social injury for them. 

In Karimi and Eskandari study 53% of runaway girls had cluster B personality disorders (31). The results of study in Isfahan showed that runaway girls and women comparing to normal group had upper scores of interpersonal hypersensivity, depression, anxiety and paranoid thinking in SCL-90 questionnaire (32). 

## Conclusion

 The results of this study revealed that girls with a history of neglect and physical abuse are prone to psychological problems leading to running away from their home. This phenomenon is significantly associated with parental addiction. High levels of extroversion, emotionality and disturbances of social skills may bring about more social injury for these girls.

## Authors’ contribution

ShT conceived and designed the evaluation, guided the research and analysis, and helped to draft the manuscript. GhRN did the literature review. GhRD re-evaluated clinical data and revised the manuscript. HSE participated in designing the evaluation, collected and evaluated the clinical data, and performed the statistical analysis. All authors read and approved the final manuscript. This paper presents the results of DrHasan Sadr Esfahani’s academic thesis.

## Notes

**Declaration of interest**: None.
